# Neurohealth Properties of *Hericium erinaceus* Mycelia Enriched with Erinacines

**DOI:** 10.1155/2018/5802634

**Published:** 2018-05-21

**Authors:** I-Chen Li, Li-Ya Lee, Tsai-Teng Tzeng, Wan-Ping Chen, Yen-Po Chen, Young-Ju Shiao, Chin-Chu Chen

**Affiliations:** ^1^Grape King Bio Ltd, Zhong-Li Dist., Taoyuan City, Taiwan; ^2^Institute of Biopharmaceutical Sciences, National Yang-Ming University, Taipei City, Taiwan; ^3^Institute of Food Science and Technology, National Taiwan University, Taipei City, Taiwan; ^4^Department of Food Science, Nutrition and Nutraceutical Biotechnology, Shih Chien University, Taipei City, Taiwan; ^5^Institute of Biotechnology, National Changhua University of Education, Changhua, Taiwan

## Abstract

*Hericium erinaceus*, an ideal culinary-medicinal mushroom, has become a well-established candidate in promoting positive brain and nerve health-related activities by inducing the nerve growth factor from its bioactive ingredient. Among its active compounds, only erinacine A has confirmed pharmacological actions in the central nervous system in rats. Hence, this review has summarized the available information on the neurohealth properties of *H. erinaceus* mycelia enriched with erinacines, which may contribute to further research on the therapeutic roles of these mycelia. The safety of this mushroom has also been discussed. Although it has been difficult to extrapolate the *in vivo* studies to clinical situations, preclinical studies have shown that there can be improvements in ischemic stroke, Parkinson's disease, Alzheimer's disease, and depression if *H. erinaceus* mycelia enriched with erinacines are included in daily meals.

## 1. Introduction

Diseases of the aging nervous system, such as Parkinson's disease, Alzheimer's disease, and stroke, are serious global public health crises as there is no cure for them currently. These lucrative markets have thus attracted the interest of a majority of large pharmaceutical companies which have put a tremendous effort into seeking medications to relieve the symptoms. However, despite successful preclinical testing, clinical trials for novel drugs have a poor track record of success.

In stroke and traumatic brain injuries, a variety of N-methyl-D-aspartate receptor antagonists have halted the progression of secondary damages in rodent models [1, 2], yet they have failed in human clinical trials due to unwanted side effects of the drugs [3, 4]. Likewise, levodopa is the primary treatment for Parkinson's disease that passes through the blood-brain barrier and gets converted into dopamine, but its long-term use can elicit additional clinical symptoms such as psychosis, mood fluctuations, increased cognitive impairment, or drug-induced dyskinesias [5]. Similarly, despite one new drug out of 244 compounds tested in 413 Alzheimer's disease clinical trials between 2002 and 2012 being approved for use, it cannot stop Alzheimer's from progressing [6]. Even though several other studies are underway, huge disappointment from the largest pharmaceutical companies, such as Axovant Sciences Ltd., Merck & Co Inc., Biogen Inc., Prana Biotechnology Ltd., and Pfizer Inc., was observed during recent times [7]. With a significant number of failed clinical trials and without a clear understanding of the potential mechanism of these diseases, dementia specialists have therefore turned their focus from treatment to prevention to stop further disease progression [8].

It is time to stop dementia before it starts. Recently, the search for small preventative neurotrophic compounds that can cross the brain-blood and are responsible for the maintenance, survival, and regeneration of neurons has attracted much attention [9]. In particular, compounds derived from natural sources with fewer side effects that can be part of everyday nutrition may help with dementia prevention. Mushrooms, which are considered nutritionally functional foods and sources of physiologically beneficial medicines, can be excellent candidates for this cause.

**Figure 1:**
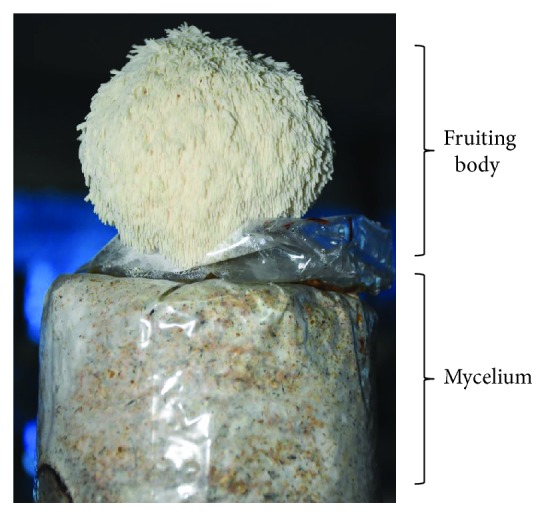
Fruiting body and mycelium of *H. erinaceus.*

Among all culinary mushrooms, *Hericium erinaceus* (most commonly known as lion's mane) has been widely reported to have therapeutic activities related to the promotion of nerve and brain health. Different compounds isolated from this mushroom inducing the expression of neurotrophic factors such as nerve growth factors (NGF) have been actively studied and reported [10–15]. Hericenones were typically found in the fruiting bodies while erinacines were derived from the mycelia of the mushroom (Figure 1).

A previous double-blinded clinical study has shown that oral administration of *H. erinaceus* fruiting body was effective in improving mild cognitive impairment in 50- to 80-year-old Japanese patients [16]. However, when examining the constituents of this effect, hericenones failed to stimulate NGF gene expression in primary cultured rat astroglial cells and 1321N1 human astrocytoma cells [17], suggesting that hericenones were not the key components responsible for the neuroprotective activities of this mushroom. On the other hand, the prominent beneficial effect of erinacine A was confirmed in the central nervous system in rats [18]. It is essential to know the concentrations of the bioactive compounds present in the functional ingredients to better assess their effects on the quality and bioactivity. For food industries, it is even critical that strict specifications of their ingredients are complied with. Therefore, this review will summarize the recent advances on the neurohealth properties of *H. erinaceus* mycelia enriched with erinacines (≥3 mg/g) and discuss the potential mechanisms of action responsible for these medicinal properties.

## 2. Erinacines

**Figure 2:**
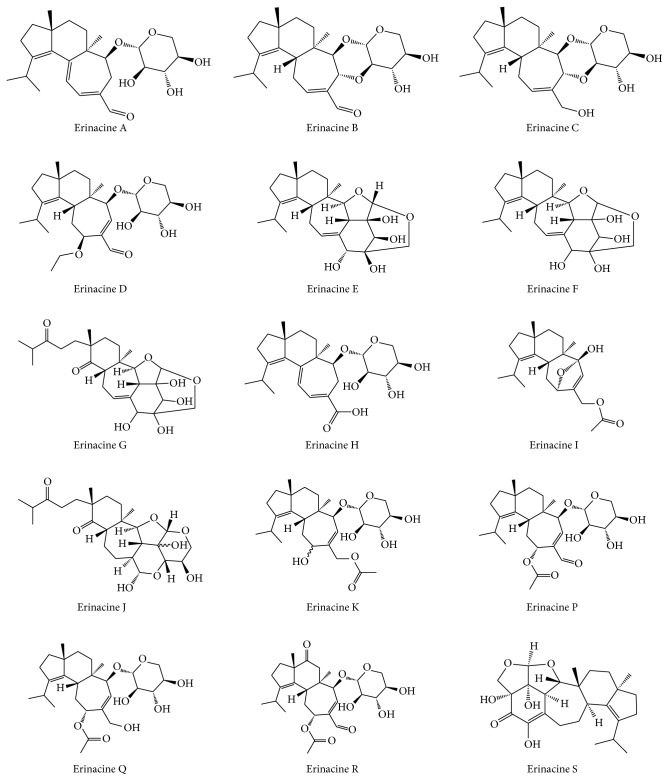
Chemical structures of 15 erinacines.

Erinacines are groups of cyathin diterpenoids that show biological activities as stimulators of NGF synthesis and could be useful as a treatment for neurodegenerative disorders and peripheral neuropathy [19]. To date, 15 erinacines (erinacines A–K and P–S) have been identified (Figure 2) and further investigations have demonstrated that eight of them have various neuroprotective properties, such as enhancing NGF release (erinacines A–I), reducing amyloid-*β* deposition, increasing insulin-degrading enzyme (IDE) expression (erinacines A and S), or managing neuropathic pain (erinacine E), while others are either being currently discovered or have other pharmacological activities (Table 1). However, no direct evidence has yet shown that these compounds could pass through the blood-brain barrier. While other bioactive agents are still being explored, erinacine A has currently been the only one designed specifically to correlate results from *in vitro* studies with outcomes observed from *in vivo* studies [18], which could bring scientists a step closer to developing a better treatment option for neurodegenerative disorders.

### 2.1. Erinacine A

Erinacine A, the main representative of the erinacine group, not only has an enhancing effect on NGF synthesis *in vitro* [12] but also can increase NGF and catecholamine content in the locus coeruleus and hippocampus of rats after administration (8 mg/kg body weight) [18]. This enhanced amount of NGF appears to markedly increase neuronal survival in different brain areas and substantially improve behavioral outcomes in various animal models. In the experimental model of stroke, 1 mg/kg erinacine A administered intraperitoneally in rats for 90 min significantly increased cell survival, attenuated the expression of proinflammatory mediators, and reduced infarct volume after transient focal cerebral ischemia [24]. In another study, it was shown that oral treatment with erinacine A could reduce amyloid-*β* plaque burden by increasing A*β* degradation by elevating the level of IDE in 5-month-old APPswe/PS1ΔE9 double transgenic mice [20]. These preclinical studies are very encouraging and suggest that erinacine A is effective in reducing neurodegenerative disease-induced cell death. However, no studies have shown that erinacine A could be absorbed into the blood capillaries, cross the blood-brain barrier, and be localized in the brain. Hence, future studies measuring the concentration of erinacine A in the brain and blood could be performed to clarify these mechanisms in detail.

Interestingly, neuroprotective compounds may also be effective in cancer therapy. Given the increasing evidence showing that genes are upregulated in central nervous system disorders and downregulated in cancers and *vice versa* [25], it suggests a bright future for developing common therapeutic approaches in the treatment of these diseases. In line with this finding, treatments with erinacine A have been found to inhibit the proliferation of DLD-1 colorectal adenocarcinoma cells *in vitro* as well as the growth of DLD-1 tumors *in vivo* [21] (Table 1). Despite the promising results, erinacines in *H. erinaceus* mycelia are usually present in microquantities and minor variations in the environment can have huge impacts on the quantity, quality, and diversity of the metabolic products.

## 3. Production of Erinacines

As the fruiting body was reported to contain no erinacines [26], the best option would be to enhance erinacine production in *H. erinaceus* mycelia via submerged fermentation under constantly controlled culture parameters. Although chemical syntheses of cyathane-type diterpenoids are not impossible, they are complex, multistep processes that result in low yields and low purity levels [27]. Therefore, it seems highly desirable to biosynthesize erinacines using bioreactors to obtain a high yield of mycelia with high concentrations of bioactive metabolites, which can expand mushroom potentialities for the development of functional foods, nutraceuticals, and novel drugs [28].

While there may have been various strategies developed over the past few decades for erinacine accumulation, it appeared, however, that only three reports concerning erinacines A and C have been published. In a 10 l bioreactor, a medium comprised of glucose 69.87 g/l, casein peptone 11.17 g/l, NaCl 1.45 g/l, ZnSO_4_ 55.24 mg/l, and KH_2_PO_4_ 1.0 g/l with a pH of 4.5 has produced 192 ± 42 mg/l of erinacine A after 8 days of cultivation [29]. With the monitoring of the temperature and ventilation during the processing, the highest yield of 206 ± 7 mg/l (17.34 mg/g) of erinacine A could be obtained after 14 days of cultivation using a 100 l bioreactor with the medium containing 0.5% yeast extract, 4% glucose, 0.5% soybean powder, 0.25% peptone, 1% oat, and 0.05% KH_2_PO_4_ at pH 5 [30]. These results suggest that a carbon-to-nitrogen (C/N) ratio of 6 and a pH value of 4 to 5 in a medium may be important parameters in promoting the biosynthesis of erinacine A in *H. erinaceus* mycelia.

Scale-up of pilot plant fermentors to large-scale bioreactors to enhance the biomass as well as erinacine production could also be an attractive proposal. Although various factors such as improper distribution of oxygen, uneven distribution of the media, or insufficient agitation environment could cause negative impacts on product formation and quality at a higher scale of operation [31], there has been one successful example of commercial exploitation. In this case, the medium was optimized for a C/N ratio of 10, temperature of 26°C, pH of 4.5, and agitation of 120 rpm. The highest accumulation of erinacine A (5 mg/g) was observed with 20-ton fermentors after 12 days [32]. This preliminary result was satisfactory, showing that implementation and successful commercial exploitation of research results in large-scale bioreactors are possible.

For erinacine C production, the optimal medium was found to include 5 g/l oatmeal, 1.5 g/l calcium carbonate, and 0.5 g/l Edamin® K at pH 7.5, which can generate concentrations up to 2.73 g/l after six days of cultivation [33]. However, it is noteworthy that this process was accomplished in a two-step course. The fungal pellets were concentrated by centrifugation to remove preculture medium components before inoculation of the main culture. Although an inoculation ratio of 5 : 10 volume/volume (*v*/*v*) is beneficial in producing erinacine C, it is only reproducible at a small laboratory scale and not feasible in industrial operations as the concentrated biomass is not easily adapted for the aseptic handling of large volumes.

These findings are extremely important as they could be used as references to enhance the production of useful secondary metabolites for industrial applications. Moreover, it should be noted that the presence of erinacines in *H. erinaceus* mycelia can also achieve pharmacological benefits. In this regard, isolation of erinacines from *H. erinaceus* mycelia is particularly important, as they could serve as quality controls in assuring the efficacy, quality, and safety of this mushroom in future markets.

## 4. *In Vivo* Preclinical Studies of *Hericium erinaceus* Mycelia Enriched with Erinacines

**Figure 3:**
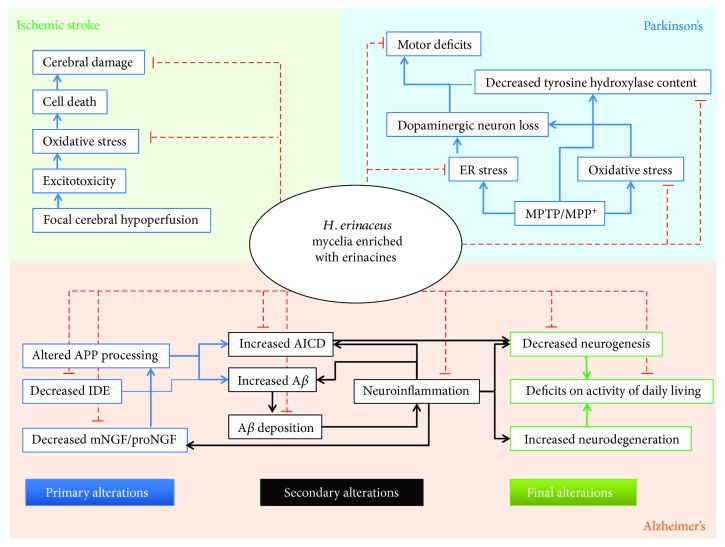
Summary of mechanisms of action of *H. erinaceus* mycelia enriched with erinacines in dementia. Primary alterations are possible contributors and drivers in the pathogenesis of Alzheimer's disease. Secondary alterations include increased amyloid precursor protein intracellular domain (AICD) and accumulation of A*β*, leading to neuroinflammation. Finally, decreased neurogenesis and increased neurodegeneration can cause deficits in activities of daily living. The red dashed lines indicate potential mechanisms of *H. erinaceus* mycelia-attenuated pathological and behavioral changes in stroke, Parkinson's disease, and Alzheimer's disease.

While 1/5 of dementia cases can be reversible in some cases when caused by drugs, alcohol, hormone imbalances, or depression, a significant proportion of individuals suffer from dementias that are irreversible [34]. The most common irreversible dementia types include Alzheimer's disease, vascular dementia, Lewy body dementia, Parkinson's disease, and frontotemporal dementia [35]. Luckily, growing preclinical studies have demonstrated that the risk of dementia and cognitive impairment could be reduced in the early stages by erinacine-enriched *H. erinaceus* mycelium consumption.  Figure 3 illustrates the overall therapeutic mechanism of action of *H. erinaceus* mycelia enriched with erinacine in dementia.

### 4.1. Protection against Ischemic Stroke

In a rat model of transient focal cerebral ischemia via the middle cerebral artery occlusion method, pretreatment with 3 mg/g erinacine A-enriched *H. erinaceus* mycelia orally at concentrations of 50 and 300 mg/kg for 5 days could reduce the total infarcted volumes by 22% and 44%, respectively [24]. Moreover, immunohistochemistry for neuronal nuclei (NeuN) revealed the presence of significantly more neurons after brain injuries in rats which were treated with erinacine A-enriched *H. erinaceus* mycelia. Excessive reactive oxygen species and oxidative stress have been strongly implicated in the pathogenesis of ischemic brain injury [36]. Decreased levels of proinflammatory cytokines and inducible NO synthase (iNOS), however, have been detected in ischemic neurons after mycelia exposure. These findings suggested that erinacine A-enriched *H. erinaceus* mycelia may be a promising agent for stroke injury as these have the ability to decrease neuronal apoptosis and reduce stroke cavity size in the rat brains by targeting iNOS/reactive nitrogen species (RNS) and p38 mitogen-activated protein kinase (MAPK)/CCAAT enhancer-binding protein homologous protein (CHOP) pathways.

### 4.2. Protection against Parkinson's Disease

Parkinson's disease (PD) is the second most common neurodegenerative disorder that is characterized by the progressive loss of dopaminergic cells in the substantia nigra pars compacta region of the brain, which results in motor problems including resting tremor, rigidity, bradykinesia, and postural instability [37]. Among models of PD, the involvement of the drug 1-methyl-4-phenyl-1,2,3,6-tetrahydropyridine (MPTP) is most widely used. Once inside the brain, MPTP is metabolized into the toxic cation 1-methyl-4-phenylpyridinium (MPP^+^) by the enzyme monoamine oxidase B, resulting in nigral dopaminergic neuronal death and mitochondrial damage, which can mimic the clinical and pathological features of PD [38]. In one study, the neuroprotective effect of erinacine A-enriched *H. erinaceus* mycelia was assessed in the MPTP-induced PD model. Results showed that dopaminergic lesions and oxidative stress in the stratum and substantia nigra were significantly improved after pretreatment with 3 mg/g erinacine A-enriched *H. erinaceus* mycelia for 25 days [39]. Furthermore, the mycelia could reverse MPTP-associated motor deficits, as revealed by the analysis of the rotarod assessment. The mechanisms underlying the neuroprotective effect of erinacine A-enriched *H. erinaceus* mycelia were associated with the inhibition on the endoplasmic reticulum stress by lowering the expression of Fas and Bax via inositol-requiring enzyme 1*α* (IRE1*α*)/tumor necrosis factor receptor-associated factor 2 (TRAF2) complex formation and phosphorylation of c-Jun N-terminal protein kinase (JNK) 1/2, p38 and nuclear factor kappa light chain enhancer of activated B cell (NF-*κ*B) pathways. Taken together, these results have demonstrated that erinacine A-enriched *H. erinaceus* mycelia have the potential to be a new therapeutic agent for the prevention and treatment of PD.

### 4.3. Protection against Alzheimer's Disease

There has been growing evidence which suggested that Alzheimer's disease progression becomes a runaway chain reaction after a certain point. In the presence of amyloid-*β* plaques, secondary injuries such as inflammation, excitotoxicity, and apoptosis may trigger the deposition of hyperphosphorylated tau proteins [40]. Once the process starts, the tau tangles are unabated even after the removal of amyloid-*β* plaques. Moreover, studies in transgenic amyloid precursor protein (APP) mice have shown that therapies are most effective when administered before plaque formation [41, 42]. Therefore, amyloid-*β* has become an ideal therapeutic target for primary prevention.

In one study, APPswe/PS1dE9 transgenic mice were utilized to evaluate the therapeutic effect of *H. erinaceus* mycelia containing 19 mg/g erinacine A on Alzheimer's disease. After 30 days of oral administration to 5-month-old transgenic mice, these mycelia were able to attenuate cerebral A*β* plaque burden, prevent recruitment and activation of plaque-associated microglia and astrocytes, promote the expression of IDE, increase the NGF-to-NGF precursor (proNGF) ratio, and enhance the proliferation of neuron progenitors and the number of newly born neurons in the dentate gyrus region [43]. Additionally, improvements in the impairment of other multiple brain regions were also shown when APP/PS1 transgenic mice treated with *H. erinaceus* mycelia could recover behavioral deficits after 81 days of administration. Collectively, these findings raise the possibility that prevention with erinacine A-enriched *H. erinaceus* mycelia could be an effective therapeutic strategy for managing Alzheimer's disease.

### 4.4. Protection against Depressive Symptoms

Depression is the most frequently occurring psychiatric comorbidity, with prevalence in Alzheimer's, Parkinson's, and stroke as high as 87%, 75%, and 79%, respectively [44]. Prior data has shown that levels of NGF are significantly lower in patients with major depressive disorder than in healthy subjects [45]. *H. erinaceus* mycelia enriched with erinacines, which are involved in the creation of the neurotrophic factors, are thereby hypothesized to play a role in depression.

In animal models, chronic restraint stress is known to cause decreased BDNF expression in the hippocampus and depression-like behaviors [46]. Hence, alleviation of *H. erinaceus* mycelia enriched with erinacines in animals subjected to repeated chronic stress was examined [47]. Two weeks of treatment with *H. erinaceus* mycelia have reduced the immobility time in the tail suspension test and forced swimming test as well as decreased the number of entries and the time spent in the open arm. In addition, restraint-induced low levels of norepinephrine, dopamine, serotonin, high interleukin-6, and tumor necrosis factor-*α* in the hippocampus were completely reversed by *H. erinaceus* mycelium administration. Furthermore, *H. erinaceus* mycelium was shown to activate the BDNF pathways and block NF-*κ*B signals in mice. Hence, these results indicate that *H. erinaceus* mycelia could be an attractive agent for the treatment of depressive disorders through the modulation of monoamine neurotransmitters and proinflammatory cytokines as well as the regulation of brain-derived neurotrophic factor (BDNF) pathways.

### 4.5. Protection against Neuropathic Pain

Currently, there is a growing realization that lesions to the peripheral or central nervous system could lead to neuropathic pain [48]. Currently, both ionotropic P2X receptors and metabotropic P2Y receptors have been identified as key receptors in mediating neuropathic pain [49]. As *H. erinaceus* mycelium has a crucial role in nerve regeneration via the stimulation of neurotrophic factors, the analgesic potential of this mycelium using both a P2 purinergic receptor-coupled Ca^2+^ signaling platform and an *in vivo* model was investigated. The results indicated that the extracts of *H. erinaceus* mycelium could completely block ATP-induced Ca^2+^ signaling in human HOS cells, suggesting its inhibitory potential as a modulator of pain-related P2X receptors [50]. In addition, administration of the extracts of *H. erinaceus* mycelium in heat-induced mice could significantly postpone the tail-flick response to heat stimulation as well as the paw-lifting response to a hot plate, indicating that it has an excellent potential for pain relief.

### 4.6. Protection against Presbycusis

Recent research has highlighted that presbycusis may precede the onset of clinical dementia and may present as an early manifestation of probable Alzheimer's disease [51]. Exogenous application of NGF has been the first to promote nerve fiber regrowth or sprouting in deafened guinea pigs caused by neomycin [52]. Moreover, clinical studies in patients with sensorineural hearing defects have revealed that the amount of circulating NGF is relatively lower compared to the level found in normal patients [53]. Therefore, the otoprotective effect of *H. erinaceus* mycelia enriched with erinacines in rapidly aging mice has been observed [54]. The results indicated that the *H. erinaceus* mycelium-treated group had significantly lower hearing thresholds according to auditory brainstem responses measured using click sounds and 8 kHz and 16 kHz tone burst sound stimulation when compared with the control group. These findings suggested that *H. erinaceus* mycelium diet supplementation was effective in slowing hearing threshold deterioration.

The beneficial activities of *H. erinaceus* mycelia on age-associated cognitive change and early dementia are summarized in Table 2. Given the fact that all seven of these studies have provided very encouraging findings, it is also of paramount importance that the daily intake of *H. erinaceus* mycelia in the context of the entire diet is established before the treatment is administered.

## 5. Toxicology Studies

To date, all experimental studies have suggested that *H. erinaceus* mycelium is safe and devoid of adverse effects (Table 3). In an animal study, the acute oral LD_50_ of *H. erinaceus* mycelia enriched with its active compounds was found to be higher than 5 g/kg in rats [55], indicating that the mycelium is reasonably safe in cases of overdose. Repeated daily doses of *H. erinaceus* mycelium enriched with its active compounds up to 3 g/kg have also been used without any adverse effects in rats [32]. Moreover, *H. erinaceus* mycelium was found not to be mutagenic in the bacterial reverse mutation test (Ames test), *in vitro* chromosome aberration test, and *in vivo* erythrocyte micronucleus test, with and without metabolic activation [56]. Further investigations also showed that erinacine-enriched *H. erinaceus* mycelium was not teratogenic in Sprague-Dawley rats with doses up to 2625 mg/kg [55]. In a well-designed clinical trial, erinacine-enriched *H. erinaceus* mycelia demonstrated significant clinical efficacy and had good safety and tolerability in 36 patients with Alzheimer's disease (unpublished data).

## 6. Conclusion

The evidence so far has shown that *H. erinaceus* mycelium enriched with its active compounds is capable of delaying neuronal cell death in rats with neurodegenerative diseases, such as ischemic stroke, Parkinson's disease, Alzheimer's disease, and depression. Moreover, results have indicated that administration of *H. erinaceus* mycelia enriched with its active compounds can promote functional recovery and enhance nerve regeneration in rats with neuropathic pain or presbycusis. Despite that more clinical research is needed to fully understand the potential applications of erinacine-enriched *Hericium erinaceus* mycelium, the majority of preclinical data strongly suggests that it is safe and offers much-needed neuroprotective applications.

## Figures and Tables

**Table 1:** Erinacines with biological activities demonstrated *in vitro* and *in vivo.*

Erinacines	Tests	Concentration	Biological activities	Reference
Erinacine A	*In vitro*	1 mM	Induced 250.1 ± 36.2 pg/ml NGF synthesis	[12]
	*In vivo*	30 mg/kg body weight/day	(1) Reduced amyloid burden by 38.1 ± 19.7% (2) Increased IDE levels by 141.1 ± 63.7%	[20]
	*In vivo*	1 mg/kg body weight/day	Inhibited DLD-1 tumor growth by 66%	[21]
	*In vivo*	30 mg/kg body weight/day	(1) Reduced both the size and number of amyloid plaques (2) Increased IDE levels by 303.5% (3) Recovered from impairments in burrowing, nesting, and Morris water maze tasks	[22]

Erinacine B	*In vitro*	1 mM	Induced 129.7 ± 6.5 pg/ml NGF synthesis	[12]

Erinacine C	*In vitro*	1 mM	Induced 299.1 ± 59.6 pg/ml NGF synthesis	[12]

Erinacine D	*In vitro*	1.67 mM	Induced 141.5 ± 18.2 pg/ml NGF synthesis	[14]

Erinacine E	*In vitro*	5 mM	Induced 105.0 ± 5.2 pg/ml NGF synthesis	[13]
	*In vitro*	IC_50_	Binding inhibitor for *κ*-opioid receptor at 0.8 *μ*M	[23]

Erinacine F	*In vitro*	5 mM	Induced 175.0 ± 5.2 pg/ml NGF synthesis	[13]

Erinacine H	*In vitro*	70.8 mM	Induced 31.5 ± 1.7 pg/ml NGF synthesis	[15]

Erinacine S	*In vivo*	30 mg/kg body weight/day	(1) Reduced amyloid burden by 40.2 ± 15.2% (2) Increased IDE levels by 130.5 ± 68.9%	[20]
	*In vivo*	30 mg/kg body weight/day	(1) Reduced the size of amyloid plaques (2) Increased IDE levels by 269.8% (3) Recovered from impairments in burrowing, nesting, and Morris water maze tasks	[22]

**Table 2:** The beneficial activities of *H. erinaceus* mycelium and its active components on age-associated cognitive change and early dementia.

Material studied (dose used)	*In vivo* models	Effects	Reference
Erinacine A	Normal Wistar rats	Enhanced NGF and catecholamine secretion in the LC and hippocampus after intragastric dosing erinacine A at 8 mg/kg body weight	[18]

Erinacine A-enriched mycelia and erinacine A	Ischemic stroke in Sprague-Dawley rats	(1) Mycelia at 50 and 300 mg/kg body weight reduced infarcted volume in cortex and subcortex of transient stroke rats (2) Erinacine A at 1, 5, and 10 mg/kg body weight reduced levels of proinflammatory cytokines such as iNOS, IL-1*β*, IL-6, and TNF-*α* in the serum of transient stroke rats	[24]

Erinacine A-enriched mycelia	APPswe/PS1dE9 transgenic mice	(1) Mycelia at 300 mg/kg body weight reduced amyloid plaque burden in the area including the cerebral cortex and hippocampus (2) Increased NGF/proNGF ratio and promoted hippocampal neurogenesis (3) Restored nesting behavior	[43]

Erinacine A Erinacine S	APPswe/PS1dE9 transgenic mice	(1) Both compounds at 30 mg/kg body weight reduced amyloid plaque burden in the cerebral cortex (2) Increased the level of IDE in the cortex by 130.5 ± 68.9% and 141.1 ± 63.7%, respectively	[20]

Erinacine A-enriched mycelia	MPTP-induced neurotoxicity in C57BL/6 mice	(1) Treatment at 10.76 and 21.52 mg/day elevated dopamine, NGF, and GSH levels (2) Reduced motor dysfunction (3) Reduced dopaminergic neurons apoptosis in the striatum and substantia nigra	[39]

Mycelia ethanolic extract	C57BL/6 mice	(1) Treatment at 2000 mg/kg body weight blocked the rise in [Ca^2+^] induced by ATP (2) Increased the latency in tail-flick and paw-lifting times exposed to a thermal stimulus	[50]

Erinacine A-enriched mycelium	Restraint stress induced depression in ICR mice	(1) Treatment at 200 and 400 mg/kg body weight increased dopamine and serotonin levels (2) Increased BDNF, Tr*κ*B, and PI3K expressions in the hippocampus (3) Reduced IL-6 and TNF-*α* levels (4) Reduced the immobility time in the tail suspension test and forced swimming test, as well as decreased the number of entries and the time spent in the open arm	[47]

**Table 3:** The safety of *H. erinaceus* mycelia.

Material studied (dose used)	*In vivo* models	Effects	Reference
Erinacine A-enriched mycelia	Normal ICR mice	No adverse effects in (1) Bacterial reverse mutation test (Ames test) up to 5 mg/plate (2) *In vitro* chromosome aberration test up to 2.5 mg/ml (3) *In vivo* erythrocyte micronucleus test up to 5 mg/kg body weight	[56]

Erinacine A-enriched mycelia	Normal Sprague-Dawley rats	(1) Ethanolic extract induced neuritogenesis in postnatal cortical neurons (2) No adverse effect up to 5 g/kg body weight/day after acute exposure (3) No adverse effect up to 2625 mg/kg body weight/day for prenatal developmental study	[55]

Erinacine A-enriched mycelia	Normal Sprague-Dawley rats	No adverse effect up to 3 g/kg body weight/day for 28 days	[32]

Based on these results, the toxicity profile of *H. erinaceus* mycelium enriched with its active compound is extremely low and therefore has the potential to be developed into a functional ingredient or food associated with improved brain and nerve health. With this idea in mind, the first erinacine A-enriched *H. erinaceus* mycelium product was introduced to the market in 2015 in Taiwan [57].

## Abbreviations

AICD: Amyloid precursor protein intracellular domain
APP: Amyloid precursor protein
BDNF: Brain-derived neurotrophic factor
CHOP: CCAAT enhancer-binding protein homologous protein
C/N: Carbon to nitrogen
ERK: Extracellular-signal-regulated kinase
IDE: Insulin-degrading enzyme
iNOS: Inducible NO synthase
IRE1*α*: Inositol-requiring enzyme 1*α*
JNK: c-Jun N-terminal protein kinase
MAPK: Mitogen-activated protein kinase
MPTP: 1-Methyl-4-phenyl-1,2,3,6-tetrahydropyridine
MPP^+^: 1-Methyl-4-phenylpyridinium
NeuN: Neuronal nuclei
NF-*κ*B: Nuclear factor kappa light chain enhancer of activated B cells
NGF: Nerve growth factor
PD: Parkinson's disease
proNGF: NGF precursor
RNS: Reactive nitrogen species
TRAF2: Tumor necrosis factor receptor-associated factor 2
*v*/*v*: Volume/volume.
